# It’s a helluva journey: a qualitative study of patient and clinician experiences of nausea and vomiting syndromes

**DOI:** 10.3389/fpsyg.2023.1232871

**Published:** 2023-08-11

**Authors:** Gabrielle Sebaratnam, Mikaela Law, Elizabeth Broadbent, Armen A. Gharibans, Christopher N. Andrews, Charlotte Daker, Greg O’Grady, Stefan Calder, Celia Keane

**Affiliations:** ^1^Alimetry Ltd., Auckland, New Zealand; ^2^The Department of Surgery, The University of Auckland, Auckland, New Zealand; ^3^The Department of Psychological Medicine, The University of Auckland, Auckland, New Zealand; ^4^The Division of Gastroenterology, Cumming School of Medicine, University of Calgary, Calgary, AB, Canada; ^5^The Department of Gastroenterology, Waitematā District Health Board, Auckland, New Zealand; ^6^The Department of Surgery, Northland District Health Board, Whangārei, New Zealand

**Keywords:** chronic nausea and vomiting syndrome, clinical care pathways, functional gastrointestinal disorders, gastroparesis, healthcare utilization, disorders of gut-brain interaction, interview

## Abstract

**Background:**

Chronic gastroduodenal disorders including, chronic nausea and vomiting syndrome, gastroparesis, and functional dyspepsia, are challenging to diagnose and manage. The diagnostic and treatment pathways for these disorders are complex, costly and overlap substantially; however, experiences of this pathway have not been thoroughly investigated. This study therefore aimed to explore clinician and patient perspectives on the current clinical pathway.

**Methods:**

Semi-structured interviews were conducted between June 2020 and June 2022 with 11 patients with chronic nausea and vomiting syndrome alone (based on Rome IV criteria) and nine gastroenterologists who treat these conditions. Interviews were recorded, transcribed, and thematically analyzed using a reflexive, iterative, inductive approach. Five key patient themes were identified: (1) the impacts of their chronic gastroduodenal symptoms, (2) the complexity of the clinical journey, (3) their interactions with healthcare providers, (4) the need for advocacy, and (5) their experience of treatments. Five key clinician themes were also identified: (1) these conditions were seen as clinically complex, (2) there is an uncertain and variable clinical pathway, (3) the nuance of investigations, (4) these conditions were difficult to therapeutically manage, and (5) there are barriers to developing a therapeutic relationship.

**Conclusion:**

Findings indicate that both patients and clinicians are dissatisfied with the current clinical care pathways for nausea and vomiting syndromes. Recommendations included the development of more clinically relevant and discriminant tests, standardization of the diagnostic journey, and the adoption of a multidisciplinary approach to diagnosis and treatment.

## Introduction

Chronic gastroduodenal disorders include nausea and vomiting syndromes (NVS; used here to encompass chronic nausea and vomiting syndrome (CNVS) and gastroparesis) (Carson et al., 2021) and functional dyspepsia (FD) (Stanghellini et al., 2016). These conditions are challenging to define, diagnose, clinically manage (Lacy et al., 2018; Tack and Camilleri, 2018; Lacy et al., 2022), and are highly prevalent, affecting up to 10% of the global population and contributing almost 30% of new patient referrals to gastroenterology clinics (Shivaji and Ford, 2014; Sperber et al., 2021). The predominant symptoms in NVS are nausea and vomiting (Carson et al., 2021), whereas FD comprises chronic early satiation, excessive fullness, and postprandial fullness (Stanghellini et al., 2016). However, there is significant symptom overlap between CNVS, gastroparesis, and FD (Camilleri et al., 2018; Pasricha et al., 2021).

An incomplete understanding of the underlying pathology, heterogeneous symptom presentation, and lack of organic biomarkers for these disorders poses challenges to clinical diagnosis (Winstead, 2011; Harer and Pasricha, 2016; Stanghellini et al., 2016; Geeraerts et al., 2020). Although several international guidelines exist for the management of these disorders, these often lack evidence-based data, resulting in controversy regarding how these conditions are defined and managed (Black et al., 2022; Drossman and Tack, 2022). An absence of multidisciplinary approaches and limited pharmacological treatments are key challenges to management (Camilleri et al., 2013). To address these difficulties, patients are often extensively investigated to exclude organic pathologies and undergo trial and error of medications and diets (Camilleri et al., 2013; Stanghellini et al., 2016; Singh et al., 2022). This results in higher healthcare utilization (Dudekula et al., 2011; Chuah et al., 2022) and is associated with more hospital admissions (Wang et al., 2008) compared to other gastroenterology patients.

Limited literature has assessed the impacts of these challenges on the clinical care pathways (McCormick et al., 2012; Rotter et al., 2012; Bennell and Taylor, 2013). Clinical care pathways are used to guide the flow of healthcare activities required for the management of a patient’s care for a given health condition (Rotter et al., 2012; Aspland et al., 2021). The care pathway refers to the journey that patients experience to access diagnosis and treatment. Anecdotal evidence posits that the clinical care pathway for patients with NVS and FD is convoluted and complex; however, there is little academic research to substantiate these claims (Basnayake et al., 2020). There is evidence to suggest that patients are dissatisfied with the current clinical care pathway due to a lack of treatment, perceived need for more testing, and frustration with the health system (Linedale, 2017). Diagnostic uncertainty also contributes to patient anxiety and frustration (McCormick et al., 2012; Woodham et al., 2022). However, a thorough understanding of the inadequacies of the current clinical care pathways and the impacts these have on the lived experiences of these conditions is unclear. Previous research corroborates the challenging lived experiences of patients with functional gastrointestinal disorders (FGIDs), including; poor health related quality of life, stigma, feelings of loss, and psychological distress (Bennell and Taylor, 2013; Woodhouse et al., 2017; Esterita et al., 2021; Taft et al., 2022). As such, efforts to better understand the clinical care pathway for patients with NVS and FD and their experiences is warranted (Peerally et al., 2021).

There is also limited literature focusing on clinician experiences of this clinical care pathway (Daker et al., 2022). Clinician perspectives of managing pediatric FGIDs highlight the need to develop strong therapeutic relationships and framing management within the biopsychosocial model (Brodwall and Brekke, 2021). Other findings suggest that primary care physicians lack confidence with diagnosing and managing adult FGIDs (Linedale, 2017). Further targeted qualitative research is needed to better understand clinicians’ perspectives of the clinical care pathways for non-pediatric populations and identify how the provision of care can be improved. This study aimed to qualitatively explore patient and clinician perspectives on the current clinical care pathways for NVS.

## Materials and methods

### Design

A qualitative interview study was conducted to gather a rich data corpus on the experiences of patients and clinicians with the current clinical care pathway for NVS. Ethics approval was granted by the Auckland Health Research Ethics Committee (AH1352). The study was conducted as per the Declaration of Helsinki and reported as per the Standards for Reporting Qualitative Research (SRQR) (O’Brien et al., 2014). All participants provided written informed consent prior to participation.

### Researcher reflexivity

The research group consisted of health psychologists, medical professionals, and bioengineers. Although the interviewers did not have a prior relationship with the patient participants, some of the researchers were known to the clinician participants. This aided recruitment; however, could have introduced bias. Interviews were conducted by a clinical academic who did not have any prior relationships with the study participants. Two health psychology researchers conducted the analysis with oversight from medical professionals and a bioengineer with specialist expertise in gastric electrophysiology. The interpretation of the thematic analysis was informed by these perspectives.

### Sample

Patients with NVS and consultant gastroenterologists who saw and treated patients with these conditions were recruited via convenience sampling. Patient participants were recruited from clinical referrals and online advertising, including via patient peer support groups. Patients were eligible for the study if they met the diagnostic criteria for CNVS (as per Rome IV criteria) (Stanghellini et al., 2016) and were aged ≥18 years. Clinicians known to see and treat these conditions were contacted to be part of the study. Maximum diversity sampling was attempted to gain a diverse clinician population. This was based on gender, age, scope of clinical practice (generalist vs. motility specialist gastroenterologists), practice type (public/private, regional/urban, academic/non-academic), and years in clinical practice. Individuals who were unable to speak or read English and vulnerable participants (e.g., individuals with a known cognitive impairment and prisoners) were excluded. Thematic saturation was reached, with no new themes identified after the 11th patient participant, and 9th clinician participant. Participant recruitment and data collection was completed between June 2020 and June 2022.

### Protocol

All participants took part in individual semi-structured interviews. These were conducted online *via* a video conferencing platform, primarily by CK, who is experienced in qualitative research methods. GS assisted with three interviews. Separate patient and clinician interview schedules were developed, based on the aims of the study, to guide the interview, with flexibility to explore any important issues that arose. Patient interviews explored symptom experience (including duration, diagnosis, management, and impact on everyday life), experience of the clinical care pathway (including experiences with healthcare professionals, testing, and treatment), and the impacts of this pathway. Clinician interviews explored their experiences diagnosing and managing patients with NVS, FD, and gastroparesis. Interviews were recorded, transcribed verbatim, and de-identified prior to analysis.

### Data analysis

Reflexive, inductive thematic analysis was conducted by two independent coders (GS, ML) (Braun and Clarke, 2006; Braun and Clarke, 2022). The two coders individually read through the transcripts to familiarize themselves with the two datasets, before grouping ideas within each dataset, into common patterns (i.e., themes). Triangulation occurred, with the two coders meeting to collaboratively develop a set of key themes with further subthemes. These themes were then reviewed by a wider research panel (CK, CNA, GOG, SC). The transcripts were reviewed against the developed themes to ensure accuracy. To generate two sets of results, patient and clinician datasets were analyzed individually. The themes are presented below in a narrative format with all themes having equal weighting.

## Results

### Demographics

In total, 11 patients and nine clinicians were interviewed. All patients met the Rome IV criteria for CNVS; however, ten also met the criteria for FD. During their interviews, eight patients mentioned that they had been diagnosed with gastroparesis, but this was not able to be corroborated clinically as researchers did not access participant medical records. The clinician sample consisted of general gastroenterologists (n = 4) and gastroenterologists specializing in gastrointestinal motility (*n* = 5). A comprehensive overview of the demographic characteristics of the sample is presented in Table 1. The clinician interviews lasted between 17 and 46 min and the patient interviews between 40 and 122 min.

**Table 1:** Demographic and clinical characteristics.

Characteristic	Patients (*n* = 11)	Clinicians (*n* = 9)
Age (years), median (IQR)	35 (25–42)	42 (39–44)
Female, *n* (%)	11 (100)	3 (33)
Ethnicity, *n* (%)		
Caucasian	9 (82)	5 (56)
Māori	1 (9)	1 (11)
Indian	1 (9)	0 (0)
Chinese	0 (0)	1 (11)
Other	0 (0)	2 (22)
Rome IV Diagnosis, *n* (%)
CNVS only	1 (9%)	–
CNVS and FD	10 (91%)	–
Specialty, *n* (%)		
General gastroenterologist	–	4 (44)
Motility specialist	–	5 (56)
Country of practice, n (%)
Australia	–	2 (22)
Canada	–	2 (22)
New Zealand	–	5 (56)
Practice experience (years), median (IQR)	–	7 (2–10)
Public practice only, *n* (%)	–	3 (33)
Public and private practice, *n* (%)	–	6 (67)
Regional practice only, *n* (%)	–	1 (11)
University affiliated, *n* (%)	–	5 (56)

IQR, interquartile range; CNVS, chronic nausea and vomiting syndrome; FD, functional dyspepsia.

### Patient themes

Five key themes, with subthemes, were identified from the patient interviews. The hierarchy of the themes is outlined in Table 2 with example quotations and briefly described in-text. Subthemes have been presented in-text using italics.

**Table 2:** Hierarchy of the themes and subthemes identified from the patient interviews with example quotations.

Key theme	Subtheme	Quotes
Impact of chronic gastroduodenal symptoms	Physical impacts	“I do not take the dog for as many walks as I used to take it for… I do not have energy to do stuff.” (P01)
	Psychological impacts	“If I’m in pain or I’m nauseous, I’m grumpy. So that has an impact on everything.” (P04)
	Relationship impacts	“In relationships… I find it’s quite isolating because I often cannot eat the same food as people.” (P09) “I’ve got really good family support, you know, and my flatmate is actually someone I went to high school with, so that’s quite helpful.” (P01)
	Employment impacts	“My managers really did understand, but it got to a point where the doctors at the hospital deemed me medically unfit for work.” (P02) “It has cost me quite a few promotions and positions I know I would have got… because I cannot work full time.” (P01)
	Effect on relationship with food	“I’m not scared of food, I’m just scared of being sick every time I eat food.” (P02)
	Use of coping strategies	“I guess I’ve learnt to live with it, it’s been going on for a while now. What can you do but adapt?” (P11) “If I manage to do my yoga regularly it kind of works as a preventive… There are times where I might start to feel it coming on, but if I start my yoga class, then by the end of the yoga class it’s gone, I feel much better.” (P10)
Complex clinical journey	Journey goes in circles	“There definitely needs, I think, a better diagnosis process because… you go around in circles for months and months, and no one can give you an answer.” (P01)
	Difficulty accessing diagnostic answers	“I feel like I have to go in prepared for battle. So I’m really expecting to fight, I’m ready to fight.” (P11) “I just felt so broken down and so upset because I’ve been here so many times and I know that it’s just an uphill battle that you really cannot win because they are the ones with the power to order the test.” (P08)
	Benefit finding through journey	“I’m happier now than I was… My mind is in a better place now than it ever was when things were seemingly going well.” (P11)
Interactions with healthcare providers	Different healthcare providers have different roles	“He [general surgeon] does not hold onto patients as long as specialists do. You kind of go and see him, you get what you need, and then you get discharged… You’ve got to go through your GP first to get the referral to go back and see the specialist. It’s not the most ideal situation.” (P11)
	Communication	“I find it really hard to get answers. You have a scan or test done in public and you do not get the results, you do not know what it is.” (P01)
	Variable clinician engagement	“I’m a wreck because they think I’m making it up… I basically did not get any care, apart from my GP.” (P03) “The test said that I had severe gastroparesis… He [surgeon] did have a look at his computer and then… pretty much just pushed us out the door. So it’s been a really rough, tough time” (P05)
	Felt inappropriately labeled	“Had the appointment with him [psychiatrist] and he accused me of having an eating disorder.” (P03) “Before the diagnosis a lot of doctors were like ‘oh no it’s in your head blah blah’. And I said ‘no it’s not’.” (P02)
Need for advocacy	Patient advocacy	“I’ve always just learned to be an advocate for myself because that’s the only way doctors can really get through to you if you stand up for yourself.” (P02)
	Clinician advocacy	“The psychologist, she worked very closely with me and told me what was going on and that she had stuck up for me basically.” (P05)
	Family advocacy	“My partner’s a really good advocate for me.” (P02) “I do not really talk openly about my health with many family members and it’s kind of a challenging one… I was raised with the attitude of like you just got to get on with life and often the word hypochondriac was thrown around.” (P11)
Experiences of treatment	Trial and error therapy	“So you name it, we have tried it.” (P04)
	Inadequacies of treatment	“I think not having treatment is the worst part.” (P02) “We’d tried Metoclopramide, all those prokinetics to get things going and they were just useless.” (P08)
	Inaccessibility of treatment	“The diet, the prescriptions, the appointments get expensive… No insurance company wants to take you on board.” (P02)
	Concern treatments/surgery are more trouble than they are worth	“I have an allergy to every single freaking antiemetic except Ondansetron.” (P03) “The pharmaceuticals I’m putting into my body, I’m really cautious about them. I’d much rather treat everything naturally and herbistically.” (P11)

#### Theme 1: Impact of chronic gastroduodenal symptoms

The first patient theme reflected the various impacts that chronic gastroduodenal symptoms had on patients’ lives. Firstly, patients mentioned the *physical impacts* of their symptoms and discussed how symptom experience limited their day-to-day physical functioning. Symptoms also resulted in *psychological impacts*, by negatively affecting patients’ mood and mental state, and *impacted their relationships with others*. Eating and drinking are central to socialization, which leads to isolation for patients who cannot partake in these activities. Patients discussed feeling like they were a burden on their family and friends. However, their experiences also revealed the strength of their relationships and highlighted the quality of their support system. Patients discussed the *impact that their symptoms had on their employment opportunities,* commenting on the difficulties they had with work and/or study. Lastly, the patients’ symptoms *impacted their relationship with food,* with patients mentioning an avoidance of food and fear of eating.

To minimize these impacts, patients employed *the use of coping strategies.* They did not want their disease to control their life, so instead found ways to learn to live with their symptoms. There was heterogeneity in the coping strategies used, with each patient finding an approach that worked for them (e.g., preparing meals, limiting physical activity, using cannabis, or doing yoga).

#### Theme 2: Complex clinical journey

The second patient theme explored the complex clinical journey patients experienced while trying to get a diagnosis and treatment for their symptoms. Their journey was not straightforward and instead appeared to *go in circles*. This journey was prolonged by a series of exhaustive tests and long wait times, which resulted in a feeling of not getting anywhere. Patients were also often sent back and forth between different healthcare providers, further convoluting their pathway. Because of this, patients found that they had *difficulty accessing diagnostic answers.* Patients reported that their healthcare providers did not always take their concerns seriously. Unfortunately, resulting in patients perceiving seeking healthcare as a battle, which sometimes led to an avoidance of accessing healthcare. However, patients did experience *benefit finding during their journey.* Having a diagnosis removed uncertainty and helped narrow down treatment options. The extensive testing, although prolonged, provided reassurance that other medical concerns had been ruled out. Some patients also showed personal growth from their experiences, discussing how they were proud of their body and that their journey had forced self-discovery and learning to find joy.

#### Theme 3: Interactions with healthcare providers

The third key patient theme discussed the extent and quality of the interactions that patients had with their healthcare providers. Throughout their journey, patients had interacted with many *different healthcare providers who played different roles in their care*. Across these providers, patients found that a lack of *communication* was an important determinant for the quality of their interactions. Patients often felt like they were not always told what was happening and it was hard to get answers. In contrast, patients highlighted the importance of receiving support and clear communication with their healthcare providers. Alongside this, healthcare providers were seen as having *variable engagement.* Although some were seen as helpful and supportive, patients discussed experiences of providers appearing to dismiss their concerns or not take their symptoms seriously. There was concern that this lack of engagement might result in them not being tested or treated for the actual issue. This view stemmed from the fact that patients *felt inappropriately labeled*. This resulted in patients being referred to specialists that were unable to help with their gastrointestinal symptoms (e.g., eating disorder clinics, gynecologists, or psychologists), further convoluting their diagnostic and treatment pathway.

#### Theme 4: Need for advocacy

The fourth key patient theme reflected the need for advocacy in their care. *Patients felt the need to self-advocate* for testing and treatment and believed they had to fight so their concerns would be taken seriously. Patients also discussed the importance of *clinician advocacy*. Some patients had experience with healthcare providers that had advocated for them in the past and mentioned the importance of this for the progression of their care. In contrast, the absence of clinician advocacy was seen as detrimental. Lastly, the importance of *family advocacy* was discussed. Some patients had family members who advocated for them; however, others experienced unsupportive family members, and felt like they had to self-advocate within their own families.

#### Theme 5: Experiences of treatment

The last key patient theme explored the experiences that patients had with treatment. Throughout their clinical journey, patients had tried many different treatments, through *trial and error therapy.* Unfortunately, patients experienced many *inadequacies of their treatments.* They found that the majority of treatments were ineffective, and wanted their disorders to be researched more so that effective treatments could be made available. Patient discussions also reflected the *inaccessibility of treatment options*, as many treatment options were difficult to access due to cost and physical availability. Lastly, there was an overall concern that *treatments were more trouble than they were worth*. Patients mentioned experiences of side-effects and allergies; while others told stories about how some treatments, such as feeding tubes, were hard for their body to tolerate. There was also concern over the long-term effects of treatments, especially when these were unknown.

### Clinician themes

Five key themes, each with a number of subthemes, were also identified from the clinician interviews. The hierarchy of the themes is shown in Table 3, with example quotations, and described briefly below.

**Table 3:** Hierarchy of the themes and subthemes identified from the clinician interviews with example quotations.

Key Theme	Subtheme	Quotes
Clinical complexity	Viewed as a complex set of disorders	“They’re a complex group… So I think because there’s that clinical challenge people feel less comfortable with it.” (C04) “Often it’s a very difficult patient group to treat, and so it does take quite a long time. And I think also that is particularly fueled by an uncertainty from the physician treating as to are they missing something or are they on the right track.” (C08)
	Difficulty of diagnosis	“It’s [Rome IV criteria] useful for trials, you need to have a standardized assessment, but when it comes to clinical practice, it’s not really all that helpful.” (C02) “I’m a bit dubious of the reliability of the gastric emptying studies that we get… Our radiologists are doing these studies infrequently and perhaps aren’t as expert at interpreting the findings.” (C07)
	Need for a multidisciplinary collaborative approach	“[I have suggested] some sort of comprehensive assessment unit analogous to the liver transplant unit; where people go for an assessment, they have a 360 assessment with all the expert assessors able to do their thing, and then provide a package of care back to the province that they come from.” (C07)
Uncertain and variable clinical Pathway	No standardized pathway to motility specialists	“I do not think we have a great framework for how we approach them… I’m sure practice varies hugely between, even within, departments, let alone between departments and between gastroenterologists.” (C09) “It’s a very variable [pathway] and I think the lack of consistency is a poor message to our patients who are already feeling rubbish… I do think a more universal approach is a really good idea.” (C04)
	Time to diagnosis varies significantly	“If someone is very classic then you can make the diagnosis on what’s right then and there… If it’s less typical, it may take a bit longer.” (C05)
Nuance of investigations	Extensive testing	“Usually they have had every test that could be done, has been done by the time they get to me… He [referring physician] does not like to leave any stones unturned.” (C07)
	Benefits of testing	“I think often the patient’s symptoms do not always improve, but it’s about being able to reassure the patient that we have actually done the right tests.” (C08)
	Inadequacies with testing	“Let us say they come and they have had no investigation, then it’s going to be months to get all the investigations done.” (C04) “If it [testing] predicted a response or helped to define a subgroup of patients then I think that sort of test would be useful.” (C06)
Difficult to therapeutically manage	Inadequate treatment options	“… severe gastroparesis or severe chronic unexplained nausea and vomiting is very difficult to treat.” (C02) “I’ve had a few [patients] who have gone off and had things like gastric electrical stimulators put in, but I have to say that the hit rate of those improving any symptom is you know about 0 %.” (C06)
	Trial and error therapy	“We just had to sort of trial and error you know… The patients get frustrated with a constant trial of medication after medication without sort of any sort of guarantee that we are actually trying the right thing.” (C08)
	Need for a multimodal approach	“I think that if it’s been determined that patients are not going to benefit from, or unlikely to benefit from, medication or interventional therapies, then I’d be very happy for psychosocial support services to take over and hopefully give patient benefit.” (C07) “I have a few dietitians, who I’ll refer people to. It’s partly for symptom control and partly for maintenance of nutrition.” (C06)
Barriers to the therapeutic relationship	Patient expectation of diagnosis	“They’ve [patients] seen a whole bunch of other specialists and you are sort of like the last port of call, and you are expected to come up with this diagnosis and treatment plan.” (C05) “Often we find that patients are looking for a label of some sort and I need to be careful about labeling somebody… until we have the [test] results.” (C02)
	Difficulty managing therapeutic relationship	“They [patients] have not had good experiences at the point that you meet them. So there’s a lot of unpicking that needs to be done.” (C04) “I think sometimes broaching some of those issues around eating disorders and psychological issues can sometimes compromise your doctor-patient relationship.” (C07)
	Clinician frustration	“I find that they require a lot of work… It’s very frequent follow ups.” (C05) “It really causes people a lot of anxiety to keep seeing the same [patient] names coming up.” (C07) “That is reversed stigma… a lot of clinicians, it seems, shudder at the thought of gastroparesis.” (C04)

#### Theme 1: Clinical complexity

The first key clinician theme explored the clinical complexity of these disorders. These were seen as a *complex set of disorders* with one clinician describing them as “a puzzle” (C08). Clinicians admitted a lack of knowledge of these disorders and noted there was little research to help guide their clinical management. Much of the complexity arose from the multifactorial, largely unknown etiology underlying these disorders, alongside the overlapping, heterogeneous, and refractory symptom presentation. Overall, the complexity of these disorders resulted in *difficulty of diagnosis*. The current diagnostic approaches (e.g., Rome Criteria and gastric emptying tests) were seen as vague, unreliable, and unable to capture the nuance of symptom presentations. Instead, clinicians relied more heavily on patient history and clinical experience. This complexity resulted in an overarching desire for a *multidisciplinary collaborative approach.* Clinicians suggested the development of a centralized, multidisciplinary service to avoid delays and wait times associated with referrals, and to allow for different specialists to work together to develop a thorough care plan.

#### Theme 2: Uncertain and variable clinical pathway

The second key clinician theme explored the uncertainty and variability in the clinical care pathway. Clinicians discussed how there was *no standardized pathway to motility specialists* and how this impacted patient care. This lack of standardization often led to delays in referral from primary care and resulted in stagnation. Clinicians noted regional and international variations in the pathway, as well as differences in the approaches of individual clinicians. This variability meant that each patient appeared to proceed to the clinician through a different pathway. This resulted in an overarching desire for a more standardized and universal pathway. Because of the lack of standardization, the *time to diagnosis for these patients varied significantly.* Most patients already had symptoms for years before they were referred to a specialist and diagnosis further took months to years from the point of referral.

#### Theme 3: Nuance of investigations

The third clinician theme explored the nuance behind diagnostic testing and investigations. Clinicians described the *extensive testing* these patients underwent; however, which tests were completed was variable and dependent on the individual provider. The clinicians further discussed the *benefits of testing* including how test results helped them exclude possible diagnoses. Testing helped inform treatment decisions and provided reassurance to patients that clinicians were making an effort towards diagnosis. However, many *inadequacies with testing* were also mentioned. For example, the accessibility of tests was variable and wait times were long. Tests were often low yield and clinicians reported a lack of reliability and standardization, especially for gastric emptying tests, which lowered their confidence in the results. Due to these concerns, clinicians iterated a need for better testing that could discriminate between different functional disorders and guide treatment decisions with more certainty.

#### Theme 4: Difficult to therapeutically manage

The fourth key clinician theme emphasized the difficulty in therapeutically managing these conditions. Clinicians highlighted the *inadequacies of treatment options* available and detailed their concerns about the lack of a “magic elixir” (C04) which was often desired or expected by patients. Management was usually done *via trial and error therapy*, where clinicians would trial different treatments to see what each patient responded to. Clinicians highlighted the *need for a multimodal approach* to management that did not just rely on medications. The importance of psychological support was discussed at length; however, clinicians noted the challenge in accessing these services and wanted more psychological support to be available. Dietary input was also seen as important to reduce symptom burden and prevent malnutrition.

#### Theme 5: Barriers to the therapeutic relationship

The last key clinician theme concerned the various barriers to the therapeutic relationship. Firstly, it was highlighted that *patients usually had an expectation of diagnosis* to validate their symptoms. Some patients seemed to come to their appointments with a predetermined diagnosis and appeared to push the clinician to confirm this (self)-diagnosis. This put pressure on clinicians to manage patient expectations and resulted in increased caution. Clinicians reported concern about giving a diagnosis too early when there was insufficient supporting evidence.

Alongside this, clinicians further discussed *the difficulty they had managing the therapeutic relationship*. Many patients had experienced previous strained clinical encounters and therefore appeared to enter the relationship with negative expectations. Reassuring these patients was seen as a challenge, especially when many did not receive a clear diagnosis or effective treatment. There was fear that this would be perceived by patients as mismanagement or lack of effort by clinicians. Lastly, this theme highlighted a degree of *clinician frustration.* Diagnosing and managing these disorders was seen as both time and resource intensive. There was an overarching feeling of frustration and anxiety with the lack of clinical improvement, despite the effort of the clinicians. These factors, alongside the complexity and challenge of these disorders, resulted in a degree of stigma and avoidance of these disorders for some clinicians.

## Discussion

The present study utilized semi-structured interviews to gain insight into the patient and clinician experiences of the current NVS clinical care pathway. Themes identified within this sample suggest that both groups were dissatisfied with the current clinical care pathway, due to perceived inadequacies in diagnosis and treatment.

Overall, patients and clinicians agreed that the current clinical care pathway is complex, uncertain, and highly variable across gastroenterologists, departments, regions, and nations. Obtaining a diagnosis and effective symptom management plan was considered time-intensive and inefficient, requiring repeated contact points with the health system, exhaustive testing, and extensive trial and error therapy. These findings conform with existing international literature (Hejazi and McCallum, 2009; Dudekula et al., 2011; Bennell and Taylor, 2013). The consistency of the present findings highlights that both groups are frustrated and are calling for improvements. For example, patients and clinicians both desired more useful diagnostic testing options to reduce the impact of the current trial and error treatment model, which is burdensome for patients and the healthcare system (Dudekula et al., 2011; McCormick et al., 2012; Moayyedi et al., 2017). Testing options that utilize actionable biomarkers could help standardize the pathway, enable clinicians to better communicate the condition with their patients, and increase consistency of care (Carson et al., 2021; Drossman et al., 2022). In turn, this could improve the therapeutic relationship and ultimately patient and clinician satisfaction (Haverfield et al., 2020).

There was also a call from both patients and clinicians for a multidisciplinary approach, which integrates gastroenterologists, dieticians, and psychologists, to manage these conditions. While patients appreciated the unique contributions of each healthcare provider within their broader care team, more cohesive coordination and communication were desired. Clinicians echoed the clinical benefits of a holistic model of care, however, noted that the current system and lack of resources often made these services difficult to access. These findings are supported by Bray and colleagues (Bray et al., 2022) who demonstrated the efficacy of a multidisciplinary, integrated treatment approach to managing patients with FD and irritable bowel syndrome. However, it is noted that caution is needed to ensure that the care pathway optimizes cohesive collaboration and serves a clinically diverse population (Richard et al., 2020).

Although patients and clinicians agreed on the complex nature of the clinical care pathway and areas for improvement, their reasons for the diagnostic and management challenges differed. This incongruence reflects a point of disconnect between patients and clinicians, which further complicates the therapeutic relationship. For example, while clinicians discussed how a fear of misdiagnosing, need for caution with labels and lack of knowledge with these conditions contributed to the lengthy diagnostic process, patients did not mention this. Instead, the long diagnostic process was perceived by patients as a lack of interest, empathy, and proactivity by clinicians. This mismatch reflects miscommunication and aligns with existing research, which explored how a lack of communication was seen as a barrier to effective care (Woodhouse et al., 2017; Richard et al., 2020).

The mismatch of perspectives, combined with the lack of effective treatments and exhaustive testing, was a source of frustration. The lack of clinical improvement, despite significant clinical input, caused frustration for clinicians and created stigma towards managing functional conditions, which further complicated care. Patients perceived the absence of clinical improvement and poor communication as their clinicians not taking their concerns seriously. This is consistent with the patients’ reported need to advocate for care, a factor which clinicians perceived added an extra layer of complexity in an already complicated dynamic. This contributed to further deterioration of an already fragile patient-clinician therapeutic relationship. Previous research supports the significance of an effective therapeutic relationship between patients and clinicians when managing chronic health conditions (Haverfield et al., 2020; Richard et al., 2020). Specifically, within patients with FGIDs, a lack of perceived empathy and communication from gastroenterologists has been found to significantly reduce patient satisfaction (Drossman et al., 2022). Therefore, it is important to improve communication between patients and clinicians to enable a positive and constructive therapeutic relationship.

While thematic saturation was achieved, some limitations are worth noting. Despite an extended recruitment period, the patient sample was predominantly Caucasian and 100% female; which may explain why referrals to gynecology were identified as a common issue. Although NVS and FD are more prevalent in females (Sperber et al., 2021), future research would benefit from exploring the experiences of males and ethnic minorities. It is notable that although an international clinician sample was recruited, the patient sample was based solely in New Zealand, limiting the patient themes to the New Zealand clinical care pathway. However, it is reasonable to assume that the current themes are likely to be representative of those countries with similar healthcare systems, such as the United Kingdom (Venkatesan et al., 2010; Richard et al., 2020).

Given the call for a streamlined multidisciplinary approach, future research could benefit from the inclusion of a broader range of healthcare providers, including, dieticians, nurses, general practitioners, pharmacists, and psychologists, who are also involved in the management of NVS. Understanding the perspectives of these providers may aid the conceptualisation of an ideal multidisciplinary model of care.

## Conclusion

The current study provides insight into the lived experiences of patients with NVS and the gastroenterologists who see and treat these patients. Both patients and clinicians experienced long, convoluted clinical care pathways, with many ongoing negative impacts. Recommendations included the development of more useful diagnostic tests, standardization of the care pathway, and the adoption of an integrated, multidisciplinary approach. Further research should aim to better understand the costs of the current clinical care pathway and therefore understand the impacts of the NVS clinical care pathway on the health system as a whole.

## Data availability statement

The raw data supporting the conclusions of this article will be made available by the authors, without undue reservation.

## Ethics statement

The studies involving human participants were reviewed and approved by Auckland Health Research Ethics Committee (AH1352). The patients/participants provided their written informed consent to participate in this study.

## Author contributions

GS, ML, EB, AG, CA, CD, GO’G, SC, and CK were involved in study conception and design. CK and GS were involved in data collection. GS, ML, EB, AG, CA, GO’G, SC, and CK were involved in the data analysis and interpretation. All authors contributed to the article and approved the submitted version.

## Funding

This study was supported by a New Zealand Health Research Council Program Grant 3715588.

## Conflict of interest

GO’G and AG hold grants and intellectual property in the field of gastrointestinal electrophysiology and are Directors in Alimetry Ltd. GO’G is a Director in The Insides Company and CK is a medical advisor for The Insides Company. GS, ML, SC, CA, and CD are members of Alimetry Ltd.

The remaining author declares that the research was conducted in the absence of any commercial or financial relationships that could be construed as a potential conflict of interest.

## Publisher’s note

All claims expressed in this article are solely those of the authors and do not necessarily represent those of their affiliated organizations, or those of the publisher, the editors and the reviewers. Any product that may be evaluated in this article, or claim that may be made by its manufacturer, is not guaranteed or endorsed by the publisher.
